# Validation of the vaccination attitudes examination scale in a South African context in relation to the COVID-19 vaccine: quantifying dimensionality with bifactor indices

**DOI:** 10.1186/s12889-023-16803-4

**Published:** 2023-09-27

**Authors:** Anita Padmanabhanunni, Tyrone Brian Pretorius, Serena Ann Isaacs

**Affiliations:** Department of Psychology, University of the Western Cape, Robert Sobukwe Road, Bellville, Cape Town 7535 South Africa

## Abstract

**Background:**

The COVID-19 pandemic represented a global public health emergency. Existing studies support the view that vaccination and mass immunization are among the most effective means of containing the outbreak and promoting health. However, negative attitudes toward vaccination and the related vaccine hesitancy among many groups have created a significant barrier to effectively managing the health crisis. Having a valid and reliable tool to assess attitudes toward vaccination remains imperative so that factors underlying vaccine refusal can be identified and public health interventions can be facilitated. The current study examined the psychometric properties of the Vaccination Attitudes Examination Scale (VAX) in South Africa.

**Methods:**

Participants (*n* = 322) completed the VAX. Confirmatory factor analysis and ancillary bifactor indices were used to examine the hypothesized factor structure (a total scale and four subscales) of the scale. Inter-item correlations, factor loadings, and average variance extracted were used to examine the validity of the scale. Predictive validity was examined by comparing those who had received the COVID-19 vaccine and those who had not. The reliability of the scale was examined in terms of both Cronbach’s alpha and composite reliability.

**Results:**

Confirmatory factor analysis provided support for the conceptualization of the scale as consisting of a total scale and four subscales, and ancillary bifactor indices indicated that the subscales accounted for a sufficient amount of variance (44%) after the variance explained by the total scale was considered. Overall, the analysis indicated that the scale had satisfactory reliability (alpha and composite reliability = 0.70) and provided evidence for the construct, convergent, and predictive validity of the VAX.

**Conclusions:**

The sound psychometric qualities of the scale, when used in a low- to middle-income country, have the potential to advance research and immunization policy within these settings and facilitate more targeted interventions to promote vaccine uptake.

## Introduction

The COVID-19 pandemic has been a global public health emergency. Existing studies support the view that vaccination and mass immunization represents the most effective means of containing the outbreak and promoting health, for example [1]. However, negative attitudes toward vaccination and the related vaccine hesitancy among many groups have created a significant barrier to effectively managing the public health crisis [2]. It is estimated that the global vaccine hesitancy rate is 25% [3]. Consequently, the World Health Organization declared vaccine hesitancy one of the 10 major threats to global health [4]. A systematic review of COVID-19 vaccination in 31 countries found that vaccine acceptance had decreased particularly in the Middle East, Africa, Russia, and Western and Eastern Europe [4]. Recent studies [5] have estimated that 25–50% of people in the United States do not intend to vaccinate despite the availability of vaccines [6].

The current study was undertaken in South Africa. The South African context offers unique opportunities for understanding vaccine attitudes, which are critical in the current global health landscape. As a major hub in international transportation [7], the country is increasingly exposed to global health risks, necessitating robust public health strategies. Importantly, South Africa serves as a microcosm for broader trends in low-to-middle-income countries (LMICs), making it a potential case for comparative analysis. Its diverse demographic profile—including varied ethnicities, socioeconomic statuses, and educational backgrounds—adds layers of complexity that are possibly beneficial for a nuanced understanding of vaccine attitudes. Furthermore, the South African healthcare system mirrors the disparities often found in LMICs, with limited resources concentrated in urban areas and significant gaps in rural regions. This uneven distribution accentuates the need to understand how different communities perceive and interact with vaccine initiatives. Additionally, the country’s influence as a regional leader in public health policy could mean that vaccine attitudes and uptake patterns observed here may set precedents for neighboring countries in Sub-Saharan Africa. Hence, understanding the psychometric properties of vaccine attitude measures in this context is not just nationally relevant but has broader regional implications. Hence, this study aims to contribute insights potentially applicable to broader public health strategies in South Africa and similar settings.

Existing studies undertaken in LMICs including those from Africa [8–11] have highlighted significant regional variations in acceptance levels. Anjorin and colleagues [12] in a survey of 34 countries in Africa reported that 63% of respondents were willing to receive the COVID-19 vaccine, 79% were concerned about side effects, and 39% were worried about COVID-19 infection after receiving the vaccine. A South African study reported an acceptance rate of 81.6%, while research from North-Central Nigeria found an acceptance rate of 29% [4]. A Ghanaian [9] study found that a fifth of adults (21%) in their sample were unlikely to receive the vaccine, while 28% were undecided. Dzinamarira and colleagues [11] reported that 20% of Zimbabweans who participated in their survey indicated unwillingness to receive the vaccine. In a scoping review on COVID-19 vaccine hesitancy in Africa, Betty and colleagues [10] reported that vaccine hesitancy was also prevalent among students and health care workers in African countries. This contrasts with studies conducted in other parts of the world (e.g., Italy, China, and France) where vaccine acceptance rates are typically higher among these groups [10].

Vaccine hesitancy appears to be context specific, but a central contributing factor is attitudinal [13–16]. A scoping review [17] of factors influencing public attitudes toward vaccination identified the following factors as salient: personal beliefs regarding vaccines, health literacy, socioeconomic status, risk perception, trust in the information disseminated by the government and media, fear of vaccine side effects, concerns about the safety and effectiveness of vaccines, and the belief that vaccines are not necessary for population immunity [17, 18].

This study is grounded in the theory of reasoned action (TRA) [19], which postulates that an individual’s intention to perform a particular behavior is influenced by their attitudes and subjective norms. The term attitude is defined as a psychological tendency to evaluate a particular situation, person, object or event in a favorable or unfavorable light [20], while subjective norms refer to the societal implications of engaging in a particular behavior [19]. Attitude contains two distinctive dimensions—namely, a cognitive component consisting of rational appraisals on a continuum of benefits or losses and an affective or emotional component [21]. A significant body of vaccination research has employed TRA as a theoretical framework and confirmed its predictive effectiveness. For example, Dube and colleagues [22] found that TRA predicted parents’ intention to vaccinate their children, while Kan and Xhang [23] reported that TRA was effective in predicting seasonal influenza vaccination behavior among the elderly.

Given the central role of attitudes in influencing vaccine acceptance and uptake as well as the significant regional variations in vaccine hesitancy in Africa, having a valid and reliable tool to assess attitudes toward vaccination remains imperative in guiding public health interventions. The Vaccination Attitudes Examination Scale (VAX) [16] was developed as a multifaceted tool to assess general attitudes toward vaccination. Validation studies on the VAX have been carried out in the United Kingdom [24], Spain [25], Italy [1], and Columbia [26], confirming the four-factor structure of the scale and providing evidence of its predictive validity. VAX adaptations have also been carried out in several countries, including Turkey [27], Romania [28], and Spain [25]. To the best of our knowledge, no studies that evaluated the psychometric properties of the VAX have emerged from sub-Saharan Africa, and this study aims to address this gap in the literature.

## Materials and methods

### Participants and procedure

Participants consisted of a random sample of students (*n* = 322) at a university in South Africa. The registrar’s office used an automated algorithm to select a random sample of 1,500 students. An electronic version of the instrument was constructed using Google Forms, and the link was sent to the selected students with an invitation to participate. The sample of 322 thus constitutes a response rate of 21.5%. The majority of the sample were women (77%), and their mean age was 26 years (*SD* = 10.2). Most of the sample were vaccinated (86.6%), and more than half of them received the vaccine as soon as their age group became eligible for it.

### Instruments

Participants completed a brief demographic questionnaire that also contained an item asking about their vaccination status, as well as the VAX scale. As the original VAX scale focused on general vaccinations, we added the following leading statement: “*The next set of statements relate specifically to the COVID-19 vaccine.”* The VAX scale consists of 12 items scored on a 6-point scale ranging from 1 (“*strongly disagree”*) to 6 (*“strongly agree”*). Higher scores on the VAX scale reflects more negative attitudes toward vaccination. In addition to a total scale score, the VAX scale also produces four subscales: mistrust of vaccine benefits (e.g., *“I can rely on vaccines to stop serious infectious diseases*”), worries about unforeseen future effects (e.g., “*Vaccines can cause unforeseen problems in children*”), concerns about commercial profiteering (e.g., “*Authorities promote vaccination for financial gain, not for people’s health”*), and preference for natural immunity (e.g., “*Natural immunity lasts longer than a vaccination*”). In the original study of the VAX scale, the authors reported reliability coefficients ranging between 0.77 and 0.93, while the relationship between the VAX scale and previous vaccination behavior, self-reported sensitivity to medicines, and the intention to obtain recommended vaccinations in the future served as evidence of validity [16]. The VAX scale has been validated in a variety of countries, and satisfactory reliability has generally been reported (e.g., Italy – *α* = 0.77 to 0.89 [29] and Spain – composite reliability (*CR)* = 0.72 to 0.82 [30]).

### Data analysis

There were no missing values, as all items were marked “must respond.” IBM SPSS for Windows Version 28 (IBM Corp., Armonk, NY, USA) was used to obtain descriptive statistics (means and standard deviations), inter-item correlations, reliabilities (Cronbach’s alpha and composite reliability [*CR*]), and average variance extracted (*AVE*). Exploratory factor analysis (EFA: principal components) was conducted to obtain the factor loadings for the total scale as well as the subscales. Prior to the EFA, we assessed whether the data was suitable for factor analysis using the Kaiser-Meyer-Olkin (*KMO*) measure of sampling adequacy and Bartlett’s test of sphericity. A *KMO* value > 0.5 and a significant Bartlett’s test (*p* < 0.05) indicates a substantial correlation among the items. In addition, IBM SPSS AMOS Version 28 (IBM Corp., Armonk, NY, USA) was used to conduct a confirmatory factor analysis (CFA) with maximum likelihood estimation to compare a one-factor model, a correlated four-factor model and a bifactor model. The following fit indices were used, as recommended by several authors [31–33]: Chi-squared (should be non-significant), the root-mean-square error of approximation (*RMSEA*: should be ≤ 0.08), the comparative fit index (*CFI*: should be ≤ 0.90), the goodness-of-fit index (*GFI*: should be ≥ 0.95), and the Tucker–Lewis index (*TLI*: should be ≥ 0.90). In addition, Arbuckle [34] recommends the use of Akaike information criterion (*AIC*) when models are being compared, and the model with the lowest *AIC* is considered a better fit for the data.

Several authors have cautioned against overreliance on model fit indices to draw conclusions about the dimensionality of a scale, as model fit indices do not address the relative strength of subscales and the total scale [35–37]. Therefore, in addition to the fit indices, ancillary bifactor indices were calculated through the use of the bifactor indices calculator [38]. These bifactor indices include explained common variance (*ECV*: the percentage of variance explained by the total scale and the respective subscales), omega (a model-based estimate of reliability) omega hierarchical (OmegaH: the proportion of variance in total scores that are the result of a single general factor), and percentage of uncontaminated correlations (*PUC*: the number of correlations between items that can be explained by a single general factor) [35]. While there are rules of thumb for each of these indices, it has been recommended that *ECV*, OmegaH, and *PUC* be considered together, rather than separately. Reise and colleagues [39] suggested that *PUC* values < 0.80, along with general *ECV* values > 0.60 and OmegaH of the total scale > 0.70, would indicate that the scale is essentially unidimensional despite the CFA fit indices supporting a bifactor structure. Finally, in terms of validity, using multivariate analysis of variance, we also examined whether the VAX was able to differentiate between those who were vaccinated against COVID-19 and those who were not.

## Results

The inter-item correlations, factor loadings, and item-total correlations for the VAX are reported in Table 1.

**Table 1:** Inter-Item Correlations, Factor Loadings and Item-Total Correlations for the Vaccination Attitude Scale

Items	Mistrust			Worries about effects			Profiteering			Natural Immunity		
	1	2	3	4	5	6	7	8	9	10	11	12
1. Feel safe after vaccination												
2. Rely on vaccines	0.8											
3. Feel protected after vaccine	0.89	0.88										
4. Vaccines may have unknown problems	0.23	0.22	0.24									
5. Vaccines can cause unforeseen problems	0.44	0.42	0.44	0.61								
6. Worry about unknown effects	0.42	0.39	0.43	0.59	0.69							
7. Vaccines make money for companies	0.37	0.41	0.42	0.37	0.5	0.4						
8. Promote vaccines for financial gains	0.41	0.46	0.45	0.34	0.54	0.38	0.76					
9. Vaccination programs are a big con	0.46	0.51	0.49	0.33	0.57	0.43	0.66	0.72				
10. Natural immunity lasts longer	0.43	0.43	0.42	0.3	0.51	0.38	0.42	0.48	0.55			
11. Natural immunity gives safest protection	0.34	0.36	0.36	0.28	0.43	0.35	0.39	0.45	0.49	0.66		
12. Being exposed to diseases naturally is safe	0.41	0.4	0.42	0.26	0.47	0.34	0.46	0.52	0.57	0.69	0.78	
Item-total *r*	0.67	0.68	0.7	0.46	0.71	0.59	0.65	0.7	0.74	0.66	0.61	0.67
Factor loadings	0.73	0.74	0.76	0.53	0.76	0.66	0.72	0.76	0.8	0.73	0.68	0.74
Subscale item-total r	0.87	0.86	0.93	0.65	0.74	0.72	0.77	0.81	0.74	0.72	0.78	0.81
Subscale factor loadings	0.94	0.94	0.97	0.84	0.89	0.88	0.9	0.92	0.88	0.87	0.91	0.92

*Note.* All inter-item correlations were significant

The inter-item correlations were all significant and ranged between 0.22 and 0.89. The highest inter-item correlations were between Item 3 and Items 1 and 2 (0.89 and 0.90, respectively). For the total scale, the item-total correlations ranged between 0.46 and 0.74, while the factor loadings ranged between 0.53 and 0.80. For the subscales, the item-total correlations ranged between 0.65 and 0.93, while the factor loadings were high, ranging between 0.84 and 0.97. With respect to the factor loadings, EFA with principal components analysis was used after KMO (*KMO* = 0.88) and Bartlett’s test (*p* < 0.001) indicated that it was appropriate to use factor analysis.

**Fig. 1:**
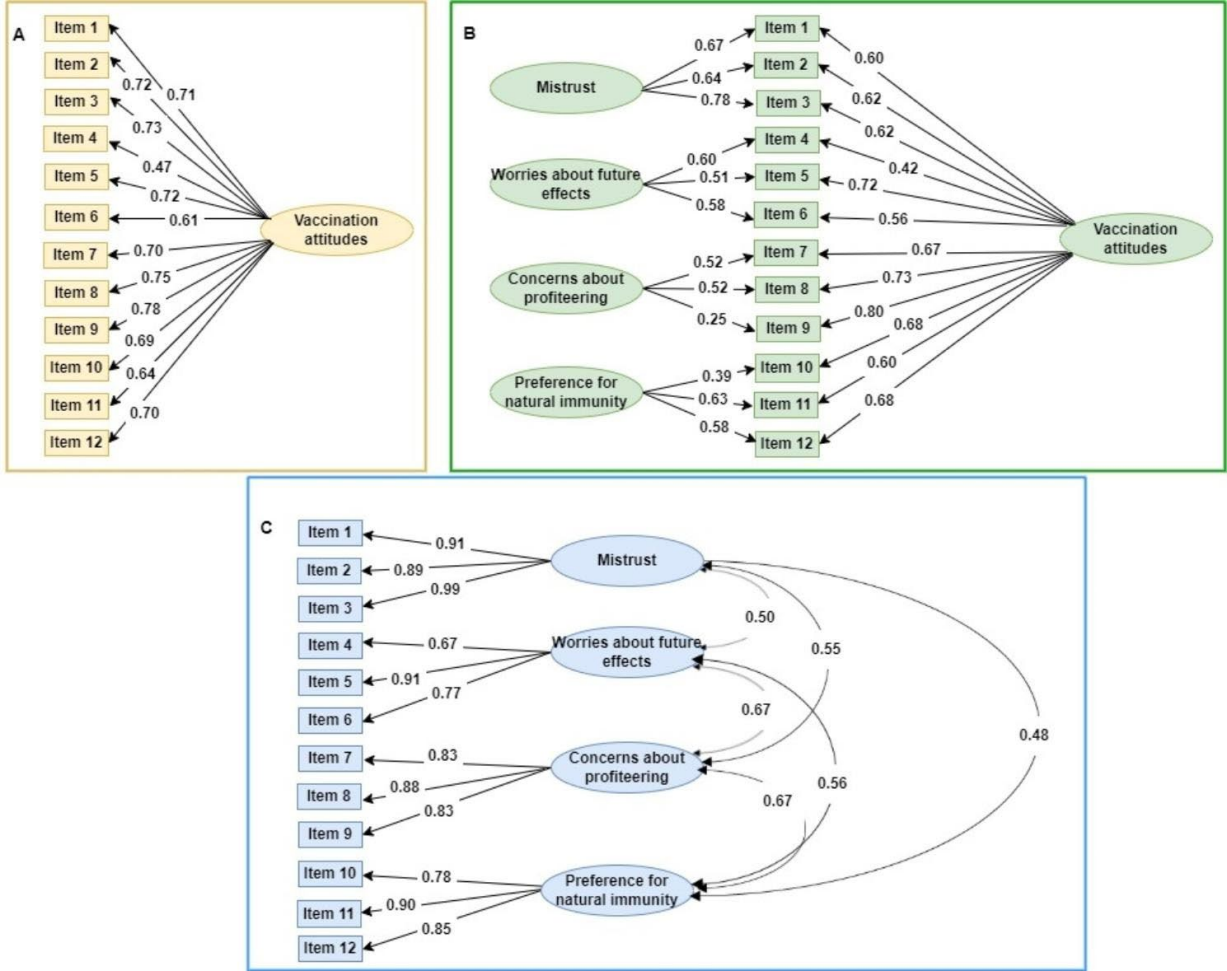
Three models of the structure of the Vaccination Attitude Scale. Note. Panel A = One-factor model, Panel B = Bifactor model, Panel C = Correlated four-factor model. Rectangles are observed variables, while ellipses are latent variables. All regression weights were significant (*p* < 0.001). See Table 1 for description of items

CFA was used to compare a one-factor structure, a bifactor structure (total scale and four subscales) and a correlated four-factor structure of the VAX scale. The three models that were compared are reflected in Fig. 1, and the fit indices resulting from the CFA are presented in Table 2.

**Table 2:** Fit Indices for Two Models of the Vaccination Attitude Scale

Goodness-of-fit indices	Best fit indicator	One-factor model	Four-factor model	Bifactor model
*χ*^*2*^(*df*)		1,205.95 (54)	101.77 (48)	44.80(42)
*p*-value	Non-significant	*p* < 0.001	*p* < 0.001	*p* = 0.35
*GFI*	≥ 0.95	0.60	0.95	0.98
*TLI*	≥ 0.90	0.51	0.97	0.99
*CFI*	≥ 0.90	0.60	0.98	0.99
*RMSEA*	≤ 0.08	0.26	0.06	0.01
*AIC*	Lower levels	1, 253.95	161.77	116.80

*Note. χ*^2^ = chi-square statistic; *GFI* = goodness-of-fit index; *TLI* = Tucker–Lewis index; *CFI* = comparative fit index; *RMSEA* = root-mean-square error of approximation; *AIC* = Akaike’s information criterion

Table 2 indicates that the fit indices for the one-factor model were not at an acceptable level (*χ*^*2*^ = significant, *GFI*: 0.60 < 0.95, *TLI*: 0.51 < 0.90, *CFI*: 0.60 < 0.90, *RMSEA*: 0.26 > 0.08). For the bifactor and the correlated four-factor model, however, all the fit indices met the criteria for good fit (bifactor model: *χ*^2^ = non-significant, *GFI* = 0.98, *TLI =* 0.99, *CFI* = 0.99 0.90, and *RMSEA =* 0.01; correlated four-factor model: GFI = 0.95, TLI = 0.97, CFI = 0.98 and RMSEA = 0.06. In addition, the model comparison index (*AIC*) was much lower for the bifactor model than either the one-factor or correlated four-factor model, demonstrating that the bifactor model is a better fit for the data than the one-factor or correlated four-factor models.

The ancillary bifactor indices are reported in Table 3. These indices reveal that specific factors (i.e., subscales) accounted for a sufficient amount of variance (*ECV* = 0.44) after considering the variance accounted for by the general factor (i.e. total scale: *ECV* = 0.56). In addition, omega reliability for the general and specific factors were all above 0.70. *PUC* > 0.80 and *ECV* < 0.60 confirms the dimensionality of the VAX and, in particular, its bifactor structure.

**Table 3:** Bifactor Indices for the Vaccination Attitude Scale

Scale	*ECV*	Omega/OmegaS^a^	OmegaH/OmegaHS^b^
VAX total	0.56	0.96	0.80
Mistrust	0.16	0.95	0.54
Worries over future effects	0.11	0.85	0.42
Concerns about profiteering	0.07	0.89	0.23
Preference for natural immunity	0.10	0.89	0.35

*Note*. percentage of uncontaminated correlations (*PUC*) = 0.82, *ECV* = explained common variance, ^a^ = Omega for total scale and OmegaS for subscales, ^b^ = OmegaH for total scale, and OmegaHS for subscales

The indices at scale level for the VAX scale are reported in Table 4. In terms of reliability, both alpha and *CR* were > 0.70 for the total scale as well as for the subscales. *AVE* in all instances were > 0.50.

**Table 4:** Indices for the Vaccination Attitudes Scale at Scale Level

Indices	Acceptable value	VAX total	Mistrust	Worries about effects	Profiteering	Natural immunity
Mean		43.8	9.3	12.9	10.9	10.7
*SD*		14.1	5.0	4.0	4.5	4.3
Alpha	> 0.70	0.91	0.95	0.83	0.88	0.88
*CR*	> 0.70	0.93	0.97	0.90	0.93	0.93
*AVE*	> 0.50	0.52	0.90	0.75	0.80	0.80

*Note*. *CR* = composite reliability, *AVE* = average variance extracted

A multivariate analysis of variance confirmed a significant overall difference between those who were vaccinated and those who were not (Hotelling’s *T*^2^ = 31.99, *p* < 0.001). In particular, the results of the univariate analyses indicated that those who were not vaccinated had significantly (*F* = 93.25, *p* < 0.001) more negative attitudes toward vaccination (Mean = 60.81, *SD* = 10.15) than those who were vaccinated (Mean = 41.14, *SD* = 12.75). Similarly, in terms of the subscales, those who were not vaccinated reported more mistrust in the benefits of vaccinations (*F* = 116.40, p < 0.001, Mean = 15.86, *SD* = 3.51), more worries about the unforeseen effects of vaccinations (*F* = 33.35, p < 0.001, Mean = 16.05, *SD* = 3.03), more concerns about vaccination profiteering (*F* = 33.16, p < 0.001, Mean = 14.40, *SD* = 4.04), and a higher preference for natural immunity (*F* = 46.70, p < 0.001, Mean = 14.51, *SD* = 3.63) than those who were vaccinated (mistrust in the benefits of vaccinations: Mean = 8.33, *SD* = 4.36; worries about unforeseen effects: Mean = 12.40, *SD* = 3.97; concerns about vaccination profiteering: Mean = 10.33, *SD* = 4.34; preference for natural immunity: Mean = 10.08, *SD* = 4.01).

## Discussion

With the global outbreak of COVID-19 and the development of vaccines to contain its spread, vaccine hesitancy among specific groups has been creating a significant barrier in effectively managing this public health emergency. Even prior to the current pandemic, the World Health Organization ranked vaccine hesitancy as one of the top 10 threats to global health [40]. This underscores the urgency of investigating attitudes toward vaccination and the related necessity of sound, cross-culturally applicable psychometric instruments to measure these attitudes. The aim of the current study was to examine the psychometric properties and applicability of the VAX in South Africa during COVID-19. Overall, the findings replicated those of the original validation study [16] but also added additional insights into the dimensionality of the scale through the use of ancillary bifactor indices.

First, the reliability of the VAX may be deemed satisfactory in terms of both Cronbach’s alpha and CR, exceeding the conventional cut-off of 0.70 [41]. Second, there were several indicators of validity. In terms of construct validity, the CFA and the ancillary bifactor indices supported the conceptualization of the VAX as consisting of a total scale score as well as subscale scores for four subscales—mistrust in the benefits of vaccines, worries about the unforeseen effects of vaccination, concerns about commercial profiteering, and a preference for natural immunity. However, while not the best fitting model the good fit of the correlated four-factor model provides support for the use of the four subscales, independently of the total scale. Further evidence of construct validity was provided by the inter-item correlations and item-total correlations. Inter-item correlations should ideally be between 0.15 and 0.85 [42]: if they are lower than 0.15, the items do not have much in common, and if they are above 0.85, the items might be redundant. In the current study, all of the inter-item correlations, with the exception of Item 3, falls within the range of 0.15 and 0.85. Item 3 was highly correlated with Items 1 and 2. However, since these three items are from one subscale that only consists of three items, the issue of the redundancy is not severe. With respect to the item-total correlations, it has been suggested that significant item-total correlations and item-total correlations > 0.50, provides further evidence of construct validity and indicates that all items make a significant contribution to the measurement of the construct [43, 44]. All the item-total correlations were significant, and only Item 4 was slightly below 0.50.

Convergent validity of the VAX was demonstrated through significant factor loadings [45, 46], AVE > 0.50, and AVE < CR [46]. The latter indices indicated that the variance explained by the scale (or subscales) is greater than cross-loadings or measurement error [47]. Lastly, the VAX was able to distinguish between those who had been vaccinated and those who had not, thus providing evidence for predictive validity.

This study has important implications. The sound psychometric qualities of the scale when used in low- to middle-income countries can aid in the advancement of research and immunization policy within these settings. The VAX has the potential to provide reliable information on attitudinal factors impeding vaccination uptake and may allow for more targeted interventions. Currently, the majority of interventions addressing vaccine hesitancy have been developed and validated in the United States and have provided inconsistent results [2]. A systematic review on strategies to address vaccine hesitancy [48] reported that the most common intervention involved education (e.g., information pamphlets or information dissemination via the media) but were largely ineffective and, in some cases, actually decreased intention to receive vaccinations. Since vaccination attitudes are influenced by culture, ethnicity, socioeconomic status, gender, and educational level [2], investigating attitudinal factors among different groups and developing tailored strategies is a necessity for the containment of future disease outbreaks. A standardized, validated measurement tool of vaccination attitudes could aid in the advancement of research in this area.

However, the study also had several limitations. First, the study used a sample comprising of university-educated students; this may have had a bearing on their attitudes toward vaccination, thus skewing the psychometric properties of the VAX. Since our sample is not representative of all sub-groups in the country, testing the scale among groups who are more at risk of not receiving vaccinations may be beneficial. Second, the use of self-report measures and an electronic survey format raises the potential for participant selection bias and social desirability bias. It is recommended that future studies include a more diverse sample and triangulated research designs. Third, it is important to consider the low response rate of 21.5%. This may limit the generalizability of the results, as it is possible that those who chose to participate in the survey differ systematically from those who did not.

## Conclusion

To our knowledge, this was the first instrument validation of the VAX in South Africa, and the study confirms that the instrument can be a useful measure in assessing vaccination acceptance in South Africa. The use of the VAX in low- to middle-income countries can potentially provide stakeholders (e.g., public health officials and governments) with relevant insights regarding attitudinal factors that influence vaccine uptake. This information can assist in the development of strategies to build trust among the public and reduce vaccine hesitancy.

## Data Availability

The data sets generated and/or analysed during the current study are available from the corresponding author upon reasonable request.
